# Not Your Common Athletic Heart Problem: Using Coronary CTA to Visualize Spontaneous Coronary Artery Dissection

**DOI:** 10.1155/2020/8882561

**Published:** 2020-10-13

**Authors:** Padma Shenoy, Taher Tayeb, Pedro Covas, Nardos Temesgen, Cynthia Tracy

**Affiliations:** The George Washington University Hospital, Washington, DC, USA

## Abstract

A 19-year-old healthy male collegiate athlete presented with typical anginal symptoms after running a 5K race. He had complained of similar symptoms off and on for the past month. On presentation, troponin was 0.12 ng/ml (reference value < 0.01 ng/ml), which peaked at 17.7 ng/ml and CK-MB was 28.71 IU/L (reference value < 25 IU/L). ECG showed diffuse biphasic T-waves. Coronary computed tomography angiogram (cCTA) demonstrated a 1.5 cm dissection in the left anterior descending artery and a 1.9 × 1.8 cm attenuation defect in the left ventricular apex consistent with thrombus. Subsequent coronary catheterization confirmed dissection of the left anterior descending artery. Spontaneous coronary artery dissection (SCAD) is a rare phenomenon. Diagnosis can be made through noninvasive measures but is usually done through left heart catheterization. In young patients who present with an NSTEMI, clinical suspicion for SCAD among other conditions should be raised. Additionally, recognizing that complications such as intracardiac thrombi can occur in SCAD is critical in ensuring appropriate therapy.

## 1. Background

SCAD is a rare, nonatherosclerotic condition known to affect predominantly women with a mean age of 42-53 years [1, 2]. It can cause acute coronary syndrome in patients who do not have typical risk factors such as hypertension, hyperlipidemia, diabetes mellitus, or tobacco use. Risk factors for SCAD include female sex, pregnancy, connective tissue disorders, fibromuscular dysplasia, physical exertion, and cocaine abuse. In some cases, cannabis use has been associated with coronary artery dissection [3]. Additionally, complications from SCAD including left ventricular thrombus have been documented [4].

In this case, the patient is a young active male who may have had a cannabis use history prior to his coronary artery dissection. It is important to include SCAD in the differential diagnosis for otherwise healthy, young, and/or male patients, especially in the setting of suspected or documented cannabis use. Patients should be evaluated for additional complications in all cases of SCAD such as mural thrombi, so that adequate therapy can be provided.

## 2. Case Presentation

A 19-year-old male collegiate athlete with no chronic illnesses presented to the Emergency Department with typical anginal symptoms after running a 5K race. He had chest tightness and sharp pain localized to his center and left chest. Associated symptoms included mild back pain and left arm tingling. He had similar symptoms for the month prior to presentation, which he would experience 15-20 minutes following a strenuous workout and lasting up to 2 hours. He presented to the hospital after his race because his pain was persistent and unremitting through the day.

His family history includes hypertension and type 2 diabetes mellitus in his parents. The patient denied history of tobacco, alcohol, or illicit drug use including cannabis and cocaine.

On arrival to the hospital, vital signs were stable with blood pressure 143/87 mmHg and heart rate of 81 beats per minute. Physical exam was unremarkable. The patient's ECG showed diffuse ST-segment changes and T-wave inversions. Troponin was 0.12 ng/ml on presentation and peaked at 17.7 ng/ml. CK-MB was 28.1 IU/L.

The patient was given aspirin, morphine, and metoprolol and admitted for workup and treatment of NSTEMI.

## 3. Investigation

**Figure 1:**
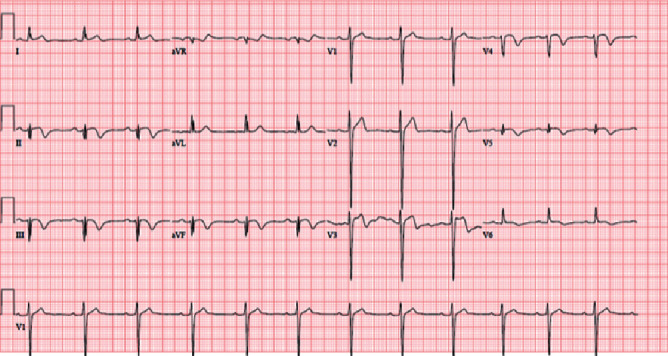
12-lead ECG showing ischemic changes.

The patient had no known conditions prior to presentation, and there had been no pre-existing workup. As mentioned above, the patient had elevated troponin levels and CK-MB on presentation. ECG showed diffuse ST changes and T-wave inversions (Figure 1).

In addition to cardiac enzymes, laboratory tests were significant for elevated C-reactive protein (CRP) of 17.4 mg/L (reference value < 3 mg/L) and urine toxicology positive for cannabinoids and opiates. Of note, patient had been administered morphine in the hospital but did not admit to cannabis use on history.

Lipid panel was grossly unremarkable with a total cholesterol of 145 mg/dL (reference value < 200 mg/dL). Hemoglobin A1c was within normal range at 5.1%. Thyroid function tests were within the normal range. Viral panel was undetectable and HIV screening was negative. Screening for connective tissue disorders with ANA and rheumatoid factor was negative.

**Figure 2:**
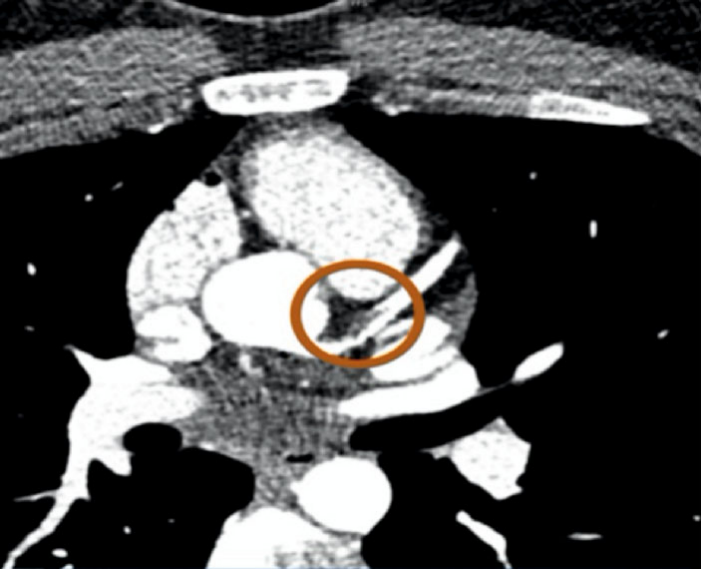
cCTA showing focal dissection in proximal LAD.

**Figure 3:**
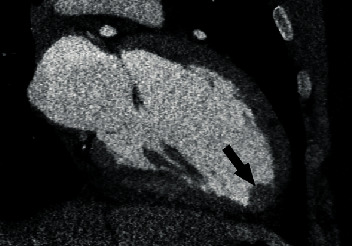
cCTA showing left ventricular apical thrombus.

Coronary CTA showed a 1.5 cm linear low-density defect in the proximal left anterior descending (LAD) artery representing a focal dissection with intramural hematoma versus adherent thrombus (Figure 2). A 1.9 × 1.8 cm thrombus was seen in the left ventricular apex (Figure 3). There was no evidence of coronary atherosclerosis.

**Figure 4:**
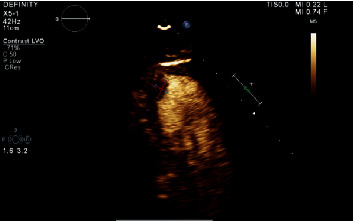
Transthoracic echocardiogram with left ventricular thrombus.

Transthoracic echocardiogram showed akinesis of apical septal, anterior, and inferior wall segments, consistent with the proximal LAD lesion. Coronary arteries were otherwise normal and without tortuosity, with an ejection fraction of 45-50%. There was a mildly decreased left ventricular systolic function with a mildly dilated left ventricle. The left ventricular thrombus was visualized and measured 1.4 × 0.7 cm (Figure 4).

Carotid ultrasound was negative for stenosis and evidence of fibromuscular dysplasia.

**Figure 5:**
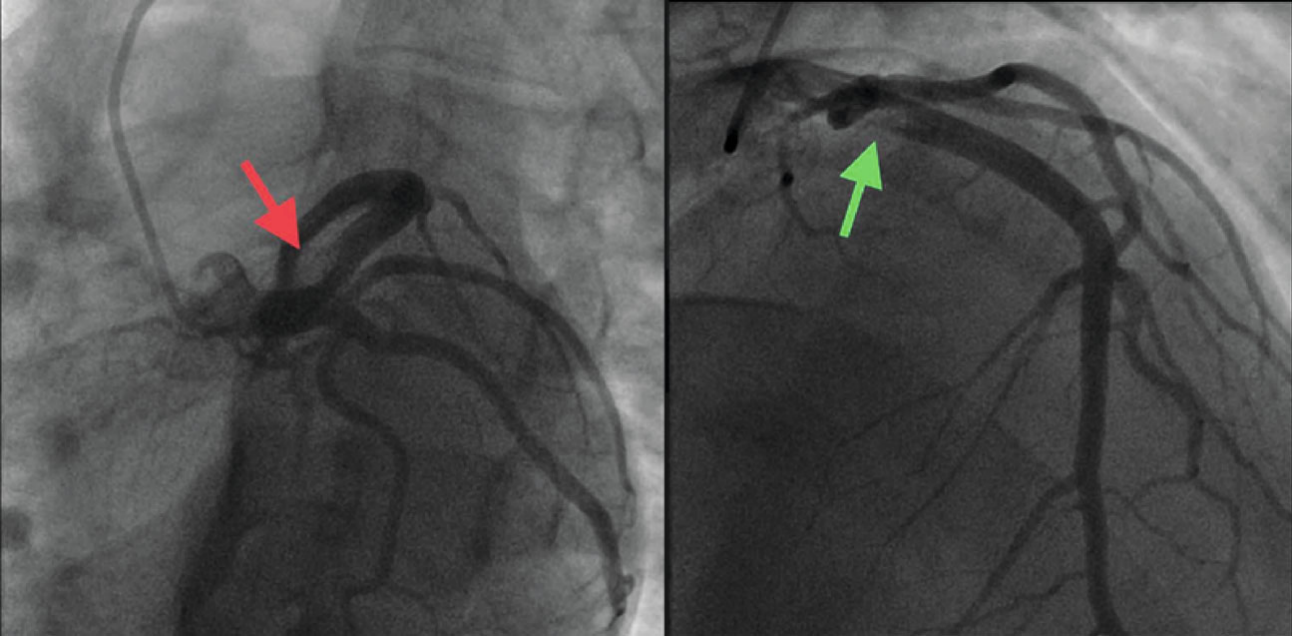
LAO caudal (red arrow) and RAO (green arrow) catheterization views of intramural filling defect of LAD.

Cardiac catheterization showed a “large caliber LAD with intraluminal filling defect in the proximal segment with residual 50% lumen. Brisk antegrade flow noted distally. There is also evidence of linear filling defect noted near the LV apex” (Figure 5).

## 4. Treatment

After imaging demonstrated an LV mural thrombus, the patient was started on a therapeutic heparin drip and subsequently transitioned to enoxaparin bridge to coumadin. For cardiomyopathy, he was treated with metoprolol, high-intensity statin, and aspirin. Troponin level trended down, and the patient was discharged with enoxaparin/coumadin, metoprolol, atorvastatin, and aspirin, as well as referral for cardiac rehabilitation and close outpatient follow-up with cardiology.

## 5. Outcome and Follow-Up

The patient was discharged 2 days after admission with no additional episodes of chest pain and reported feeling well. He was scheduled for regular cardiology follow-up, INR monitoring, and repeat cCTA after discharge.

An outpatient abdominal CTA showed no high-grade renal artery stenosis or evidence of fibromuscular dysplasia.

At his follow-up appointment with cardiology two days after discharge, lisinopril was added to the patient's regimen for treatment of cardiomyopathy. ECG showed Q waves in inferior leads and T-wave inversions in anterolateral leads.

His INR was monitored twice weekly until the patient was in therapeutic range of 2-3, at which point he discontinued enoxaparin.

Repeat cCTA 12 days after NSTEMI showed near-complete resolution of the previously seen low-density focal dissection with intramural hematoma of the proximal LAD. The narrowing of the LAD had completely resolved, and no new defects were seen in the LAD. The LV mural thrombus had decreased in size, measuring 0.5 × 0.6 cm.

At the patient's two-month follow-up with cardiology, ECG showed less pronounced T-wave inversions in anterolateral leads. Echocardiogram showed mild apical wall motion abnormality attributed to the recent infarct and no thrombus was seen. He had not experienced chest pain. Lisinopril and atorvastatin were discontinued, and metoprolol dose was decreased.

Three months post-MI, a cardiac MRI showed “apical aneurysm with apical and apical inferior dyskinesis. The LV function is mildly reduced with a calculated LVEF of 49%,” and coumadin therapy was discontinued.

## 6. Discussion

Coronary angiography remains the cornerstone in diagnosis of SCAD. With or without the aid of invasive imaging, the role of cCTA in diagnosis of SCAD has not been fully established. We report a case where cCTA aided in the diagnosing of SCAD in a patient presenting with typical ACS symptoms.

SCAD is a disease in which there is separation of the tunica intima and tunica media of the vessel wall which creates a false lumen leading to infarction. SCAD is a rare cause of ACS with an incidence in the general population of 0.1-0.4% [5, 6]. The first reported case was in a postmortem autopsy of a 43-year-old female. SCAD accounts for almost 25% of cases of infarction in women younger than the age 40 years and many cases go undiagnosed until autopsy [6–8]. Risk factors include physical and emotional stressors, pregnancy, fibromuscular dysplasia, and connective tissue disease [9–11].

Coronary angiography remains the gold standard in diagnosing SCAD with or without invasive imaging [12, 13]. Catheterization is associated with less desirable outcome in some cases with SCAD, most likely due to worsening of the dissection and difficulty accessing the lesion [5]. Currently, medical management is preferred [10, 14] unless the dissection is associated with the left main artery, continues to infarct, or causes hemodynamic instability [12, 15]. This encourages the use of noninvasive diagnostic modalities such as cCTA in stable patient to prevent risks associated with PCI such as bleeding, hematoma, and intracardiac injury. Additionally, it has been suggested that there is no difference in long-term outcomes between conservative therapy and PCI in patients with non-high-risk anatomy [16]. High-risk anatomy is defined as dissection in the left main artery or the development of dissection in two vessels.

Another consideration in this case was the use of anticoagulation for treatment of LV mural thrombus with concomitant coronary dissection. Risks of anticoagulation in this setting were extension of the LAD dissection and size of intramural hematoma; however, the benefit of preventing embolism of the thrombus causing a potentially devastating CVA in a young patient was considered as well. The patient was discharged with anticoagulation and antihypertensives with close follow-up to monitor the dissection flap and LV thrombus.

We present a case in which cCTA accurately diagnosed SCAD and LV thrombus, which was then confirmed by coronary angiogram and repeat cCTA showed resolution of thrombus and healing dissection. Given the uncommon incidence of SCAD, there are no studies done that show cCTA can be reliably used for diagnosing coronary dissection, but these would be of utility for diagnosis.

## Data Availability

The data used to support this study are included within the text of this report as well as in the cited references.

## Additional Points

*Learning Points/Take Home Messages*. (1) Current indication for stent placement in a case diagnosed with SCAD, which are left main disease, unstable patient, or ongoing infarct. (2) The role of cCTA as a potential diagnostic tool. (3) Recognizing SCAD as a case of ACS especially in a population below the age of 50.
